# Peripheral pain mechanisms in osteoarthritis

**DOI:** 10.1097/j.pain.0000000000001923

**Published:** 2020-08-19

**Authors:** Tonia L. Vincent

**Affiliations:** NDORMS, Centre for OA Pathogenesis Versus Arthritis, Kennedy Institute for Rheumatology, University of Oxford, Oxford, United Kingdom

## Abstract

There is a well-established historical observation that structural joint damage by plain X-ray correlates poorly with symptomatic disease in osteoarthritis (OA). This is often attributed to the inability to visualise soft-tissue pathology within the joint and the recognition of heterogeneous patient factors that drive central pain sensitisation. A major issue is the relative paucity of mechanistic studies in which molecular pathogenesis of pain is interrogated in relation to tissue pathology. Nonetheless, in recent years, three broad approaches have been deployed to attempt to address this: correlative clinical studies of peripheral and central pain outcomes using magnetic resonance imaging, where soft-tissue processes can be visualised; molecular studies on tissue from patients with OA; and careful molecular interrogation of preclinical models of OA across the disease time course. Studies have taken advantage of established clinical molecular targets such as nerve growth factor. Not only is the regulation of nerve growth factor within the joint being used to explore the relationship between tissue pathology and the origins of pain in OA, but it also provides a core model on which other molecules present within the joint can modulate the pain response. In this narrative review, how molecular and pathological tissue change relates to joint pain in OA will be discussed. Finally, a model for how tissue damage may lead to pain over the disease course will be proposed.

## 1. Introduction

The study of structural and symptomatic disease in osteoarthritis (OA) has been thwarted by having blunt tools on both sides.^6^ Plain radiographs (X-rays) are only able to visualise 2-dimensional radiopaque structures, so are good for assessing gross changes in bone shape, osteophyte size, chondrocalcinosis, subchondral bone sclerosis, and bone cysts. From the space between the bone ends, one is able to extrapolate cartilage thickness, but the meniscus, synovium, and inflammatory processes are invisible. For patient pain outcomes, many tools have been developed and validated over the years to assess patient- and physician-reported measures of pain, but these are limited by their subjectivity and necessarily complicated when trying to capture the quality and pattern of different types of pain at different times. The contribution of central and peripheral components of pain also varies between and within individuals and there are few routinely applied methods for their assessment. Taken together, it is hardly surprising that our ability to relate clinical pain outcomes with structural change has been challenging.

## 2. The course of pain in human osteoarthritis

The course of pain in human OA is variable. Some individuals seem to present with painful joints with little in the way of change on a plain X-ray. This is often described as early OA especially if the individual has other risk factors eg, age >50 years, obesity. Evidence that these individuals necessarily go on to develop OA is modest. The classification criteria for defining early OA are actively being explored by Osteoarthritis Research Society International (OARSI) and other relevant OA groups.^62^ Others can present seemingly for the first time after considerable damage has occurred,^21^ although on the whole, most individuals presenting after the age of 50 years and with joint pain will have damage evident on an X-ray.^56^ A few characteristics seem to be consistent: pain is usually mechanically induced in the early stages,^64^ worsening with exercise and better at rest; the pain is typically burning in nature but can be associated with sharp shooting pains especially, eg, in the base of the thumb; pain often waxes and wanes but as the disease progresses, it becomes more frequent and can occur at rest or at night (reviewed in Ref. 38). The structural course thereafter for an individual is unpredictable with only 40% of individuals progressing on a plain X-ray over 10 years.^59^ Almost all patients with a chronic peripheral drive of their pain will exhibit evidence of central sensitisation, but this may be particularly marked in some individuals where a more diffuse pain syndrome develops (reviewed in Refs. 2, 75). Other than qualitative pain questionnaires, quantitative sensory testing is the most commonly used clinical assessment tool for central sensitisation. Local and widespread sensory threshold changes after experimental stimulation eg, hot/cold, light touch, pressure, are common and may relate to patient outcome, for instance after joint replacement.^80^ Other sensitisation features include evidence of temporal summation and increased spinal reflexes.^60^ Patient-related factors such as stress, adverse life experiences, sex, gut microbiome, and comorbidities predispose to the development of central sensitisation and contribute to the pain phenotype.^7,24,93^

## 3. The course of pain-like behaviour in rodent osteoarthritis

**Figure 1.**
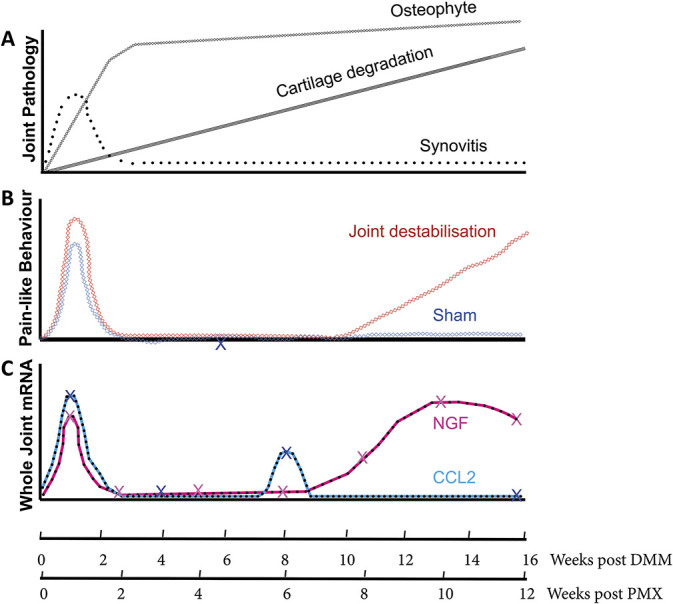
Temporal relationship between pain-like behavior, pathology, and molecular expression in murine osteoarthritis. (A) Pathological changes in the joint over the course of surgically induced OA. Initial postoperative synovitis that is modest thereafter; progressive chondropathy; rapid early osteophyte formation that grows slowly over time. (B) Pain-like behavior after surgically induced OA (red) after either DMM or PMX or sham (gray) surgery. Postoperative behavioral change in joint destabilized groups is modest compared with sham. Some groups show postoperative mechanical allodynia that persists in the destabilized group compared with sham for 12 weeks (not shown). (C) Two key pain-sensitizing molecules with proven analgesic effects in vivo and where temporal data are available on their expression, nerve growth factor and CCL2. X represents actual time points tested. Taken from Refs. 12, 26, 67,70,72,100. DMM, destabilisation of the medial meniscus.

In rodents, pain cannot be measured directly but is inferred from “pain-like behaviour.” This can be through evoked behavioural responses (von Frey hair induced mechanical allodynia, hot/cold hypersensitivity, for instance) or by studying spontaneous behavioural responses (activity levels or by distribution of weight through the diseased compared with nondiseased joint). A number of different induced and spontaneous models in small and large animals have been used to study pain behaviour in OA (comprehensively reviewed in Ref. 69). In the past 10 years, these have largely been in rodents and broadly divide into those induced by monosodium iodoacetate (MIA) and those that are induced by surgical destabilisation of the joint (destabilisation of the medial meniscus [DMM], partial or complete meniscectomy, and anterior cruciate ligament transection, alone or in combination with ligamentous injury). A small minority of studies have used spontaneous rodent models of OA: in genetically susceptible strains, by ageing animals and by dietary modification, eg, high-fat diet. Monosodium iodoacetate has been favoured historically by pain biologists because it produces rapid, robust changes in pain-like behaviour, in line with the rapidly destructive inflammatory changes in the joint. Although it was recognised that features of MIA-induced disease may be relevant to some aspects of human OA, in 2012, the Arthritis Research UK animal models working group recognised that surgical models were generally becoming favoured for pathogenesis studies because these produce insidious, progressive, relatively noninflammatory changes in the cartilage, bone, and synovium, similar to the human condition^99^ (Fig. 1A). Whether surgically induced joint destabilisation is representative of all types of OA is subject to intense debate. Despite attempts to categorise OA into different phenotypes (metabolic, post-traumatic, inflammatory, for instance), there is little evidence thus far to support a clinical benefit of doing so. In the case of synovitis, this is discussed further below. Conversely, there is a growing body of evidence showing that target discovery in surgical models of OA has clinical relevance in human nontraumatic disease; thus, these models seem to be generalisable.^23,55,67,108^

Focusing solely on surgical models, 2 distinct pain-like behaviours can be discerned: an initial postoperative phase that occurs in both OA and sham-operated animals and a late phase that, in our hands, starts at around 11 weeks after DMM, and 8 to 9 weeks after partial meniscectomy (PMX)^26,39,100^ (Fig. 1B). Evoked and nonevoked measures, at the level of the joint or distal to the joint, seem to mirror one another when they are performed in the same study.^26,48,90^ Differences in the model used to induce OA, the periodicity and length of time of follow-up (many looking only 2-4 weeks after surgery), and the behavioural tests deployed make it hard to reach a consensus on the pattern of pain-like behaviour after joint destabilisation; some groups reproducing our observed biphasic behavioural response,^26,48,72,90,100^ and others detecting mechanical allodynia early after surgery, which fails to resolve over time.^8,31,43,70,107^ Regardless, the ability to map pain-like behaviour in a temporal fashion is important because it provides an opportunity to relate this to pathological tissue changes occurring in the joint and associated dorsal root ganglia (L2-4). Studies are further enhanced by being able to perform molecular analyses on microdissected tissues of the joint, and to do such studies in genetically modified mice.^12,70,72^

## 4. Nerve growth factor as a pain target in human and murine osteoarthritis

In 2010, in a candidate molecule approach, we identified nerve growth factor (NGF) as a pain target in murine OA after DMM.^67^ *Ngf* mRNA was upregulated in the whole joint during the 2 phases of pain behaviour (postoperative and late OA pain). The drivers of NGF in each case were shown to be different; postoperative pain being inhibited by neutralising tumour necrosis factor (TNF), and the late phase seemingly independent of inflammation. Soluble tropomyosin receptor kinase A, the receptor for NGF, was able to suppress both postoperative and late OA pain-like behaviour.^67^ More recently, we also showed suppression of pain-like behaviour in mice following PMX after vaccination with murine Ngf.^100^ Other groups have also demonstrated NGF-dependent pain-like behaviour in rats including by showing that intra-articular injection of Ngf exacerbates pain-like behavioural change,^3^ and suppression of early pain-like behaviour with anti-Ngf^53^ or by inhibition of Trka^77^ after meniscal injury.

The first clinical Phase II trial of anti-NGF in patients with knee OA was published in 2010.^55^ Since then, a number of studies using neutralising antibodies have demonstrated clinical efficacy validating NGF as a target in OA pain.^86,91^ Concerns about the risk of rapidly progressive OA may yet limit its clinical utility (reviewed in Ref. 54). The FDA is yet to pronounce whether this class of drug, which was given “fast-track designation” in 2017, will be approved for use in patients in the United States. Two studies using TrkA inhibitors have also been published recently. One showed limited success after intra-articular delivery,^51^ and a second oral agent study failed to reach its primary endpoint.^104^ Neither agent confirmed direct target engagement.^102^

The success of anti-NGF in the treatment of painful OA raises several questions: Where is NGF made? What are the drivers of NGF in the OA joint? And how does this change over the course of disease? Several developments in the past few years have helped to unravel this relationship in OA: (1) use of improved imaging modalities, particularly magnetic resonance imaging (MRI), to visualise the soft tissues of the joint, (2) agnostic molecular profiling of human OA tissues, and (3) careful dissection of molecular processes occurring over the course of rodent OA. In the next section, pathological changes in each of the main tissues of the joint (synovium, meniscus, bone, and cartilage) will be considered.

## 5. The role of the synovium in osteoarthritis pain

The synovium has always been a good candidate tissue as a generator of pain responses in the OA joint; it is highly innervated, remodels in a dynamic fashion, and has a key role in driving both pain and joint damage in rheumatoid arthritis. Rheumatologists recognise joint space tenderness, joint effusion, and synovial thickening in individuals with OA, and most patients, at least anecdotally, gain benefit from intra-articular steroid. In recent years, visualisation by enhanced and nonenhanced MRI and ultrasound has enabled correlative studies with patient symptoms to be performed. Contrast-enhanced MRI is superior over nonenhanced imaging, and demonstrates that around 90% of individuals with symptomatic disease will show evidence of synovial enhancement indicative of hypertrophy and/or synovitis.^85^ Most of these studies report a positive correlation, albeit often weak, between patient symptoms and severity of synovitis.^36,109,112^ Synovitis has been shown to be predictive of disease progression in hand OA,^35,49^ and a change in volume of the synovium is associated with a clinical improvement in patients given intra-articular steroid.^78^ However, these studies have recognised limitations. They have frequently looked at synovitis separately from other pathologies in the joint, and where other pathologies have been examined, these usually co-correlate making it difficult to exclude whether synovitis is simply a biomarker of OA severity.^110^ The incorrect conflation of correlation with causation and the assumption that synovial volume necessarily means “inflammation” further complicates interpretation. After the success of disease-modifying drugs in rheumatoid arthritis, it was assumed that the same anti-inflammatory drugs would work in OA also. The recent lack of clinical success with a number of agents including anti-IL1, anti-TNF, and hydroxychloroquine indicate that this was not correct.^14,15,45–47,57,96^ Other studies including those targeting IL6 or using methotrexate have been conducted but are yet to report.

Glucocorticoids, either delivered intra-articularly or orally, are assumed to target the synovium primarily, although it is worth remembering that several tissues of the joint express glucocorticoid receptors, including chondrocytes.^92^ Randomised controlled trials (RCTs) using intra-articular or oral steroid show that the clinical response is mixed. Recent studies show that a long-acting intra-articular steroid preparation, FX0006, did demonstrate statistically significant superiority over placebo, although the Phase II study failed to meet its primary endpoint (improvement over placebo at 12 weeks).^16^ An RCT of intra-articular steroid in hand OA, in which lignocaine alone was used as a placebo, met 2 out of 11 coprimary endpoints.^88^ A recent RCT of 10-mg daily oral prednisolone did show significant improvement over placebo for the duration of treatment, reverting back to pretreatment levels of pain within 3 weeks.^50^ Few studies have examined the effect of repeated intra-articular steroid in patients with OA. In the study by McAlindon et al., 3 monthly triamcinolone intra-articular injections to individuals with knee OA over 2 years not only failed to register symptomatic improvement over placebo at any of the 3 monthly assessments, but also showed a small but significant increase in cartilage degradation in repeat steroid injected joints. This study may have missed a transient analgesic response to the steroid injection occurring within the 3-month follow-up period. Similar data have been observed in the Osteoarthritis Initiative, collectively highlighting the potential risks associated with this commonly delivered treatment.^65,111^ Most of the above studies also had imaging outcomes that showed inconsistent responses when examining synovial volume and vascularity.

Molecular studies in OA synovium come from synovial biopsies performed in individuals with OA discordant for pain. In one study, individuals with Kellgren and Lawrence (K&L) grades of greater or equal to 3 were stratified by high or low pain scores (by visual analogue scale). A microarray study was performed on synovial biopsies (from 5 individuals in each group), which revealed a number of dysregulated genes.^10^ Of the regulated genes, candidate molecules of interest that have been implicated in OA pain included *TRKB*, the receptor for brain-derived growth factor, angiotensinogen, and netrin-1. Blockade of the angiotensin type II receptor is analgesic in individuals with postherpetic neuralgia and in murine OA (see below), and netrin-1 has been shown to drive axonal growth in the subchondral bone of murine OA.^33,83,113^ There were few classic inflammatory genes regulated apart from CCL14 and ADAMTS15. Notably, *NGF* was not identified as a regulated gene. Two other published studies have looked at synovial biopsies from symptomatic and asymptomatic groups that were matched for chondropathy score. It is worth noting that in this study, the asymptomatic group was taken from postmortem samples where there was no apparent history of OA pain from first-degree relatives or patient notes. Tissue morphology was examined and candidate molecules (NGF and CD68) were examined by immunohistochemistry. This study showed that there was increased synovial hypertrophy, cartilage damage, and both NGF and CD68 immunostaining in diseased tissue from those with higher pain.^89^ A second study, by the same group, examined mRNA for 96 genes associated with pain in discovery and replication cohorts. Validated, regulated genes were further explored by protein assays. Few genes were consistent across groups, most likely reflecting the heterogeneity of patient samples, and there was a notable lack of inflammatory cytokines and *NGF* regulation even when compared with nonarthritis control tissue (presented in supplementary data).^105^ Nonetheless, some molecules showed strong or consistent regulation at mRNA and protein level including angiotensin-converting enzyme (ACE) (increased), IL1R1 (decreased), CCL2, CCL8 (increased), and MMP1 (increased). Of these, ACE and CCL2 may be of particular interest. Angiotensin-converting enzyme is involved in the conversion of angiotensinogen to angiotensin II, which can activate the type II receptor, present on pain fibres, to reduce the threshold for firing.^1,34^ Our studies show that ATII blockade is analgesic in murine OA (Vincent et al., manuscript in preparation). CCL2 has also been implicated in pain sensitisation in murine OA, causing increased macrophage infiltration in the dorsal root ganglia and delaying pain-like behaviour development.^70,72^ The notion that synovitis may drive inflammatory priming of pain, contributing to pain severity is attractive (Fig. 1C). Of interest, synovitis correlates with sensitisation, measured by quantitative sensory testing in patients^76^ and these predict response to nonsteroidal anti-inflammatory drugs.^81^

The role of an activated innate immune system in OA is strong from a variety of descriptive studies (some agnostic),^17,71,73^ but there have been surprisingly few studies that have identified immune cells specifically driving this process and contributing to pain. One exception to this is the established role of mast cells in OA pain. Mast cells are increased in murine and human OA joints and contribute to structural degradation.^11,22,103^ Mast cells also express TRKA, are responsive to NGF, and secrete NGF,^37,58^ and hence have a potentially important role in structural and symptomatic OA.

## 6. The role of the meniscus, ligaments, and other soft tissues of the joint

The meniscus and ligaments (and extra-articular muscle) are critical for maintaining stability of the joint, and damage to any one of these increases the risk of OA.^25^ This is seen upon acute ligamentous injuries such as anterior cruciate ligament rupture; around 50% of such individuals develop OA within 10 years.^61^ Extrusion of the meniscus, with or without evidence of previous meniscal tear, is also a common feature in age-related OA, suggesting that meniscus-dependent mechanical joint instability may contribute to OA risk in the elderly.^27,29,63^ Significant pathology and molecular change has been documented in human meniscus in OA or after injury,^82^ and also after joint destabilisation in rats.^28^

Much less is known about the role of the meniscus, ligaments, and other soft tissues in OA pain. Normal anatomical studies reveal that these tissues are highly innervated and this innervation can be deranged after development of disease.^4,44,66^ In the mouse, 16 weeks after destabilisation of the meniscus, there is a marked increase in calcitonin gene related peptide-positive nerve fibres associating with the medial meniscus and synovium, in addition to the subchondral bone (discussed further below),^79^ perhaps indicating that these tissues contribute to pain in OA. More studies are required in this area to be able to make more informed conclusions.

## 7. The role of the bone in osteoarthritis pain

**Figure 2.**
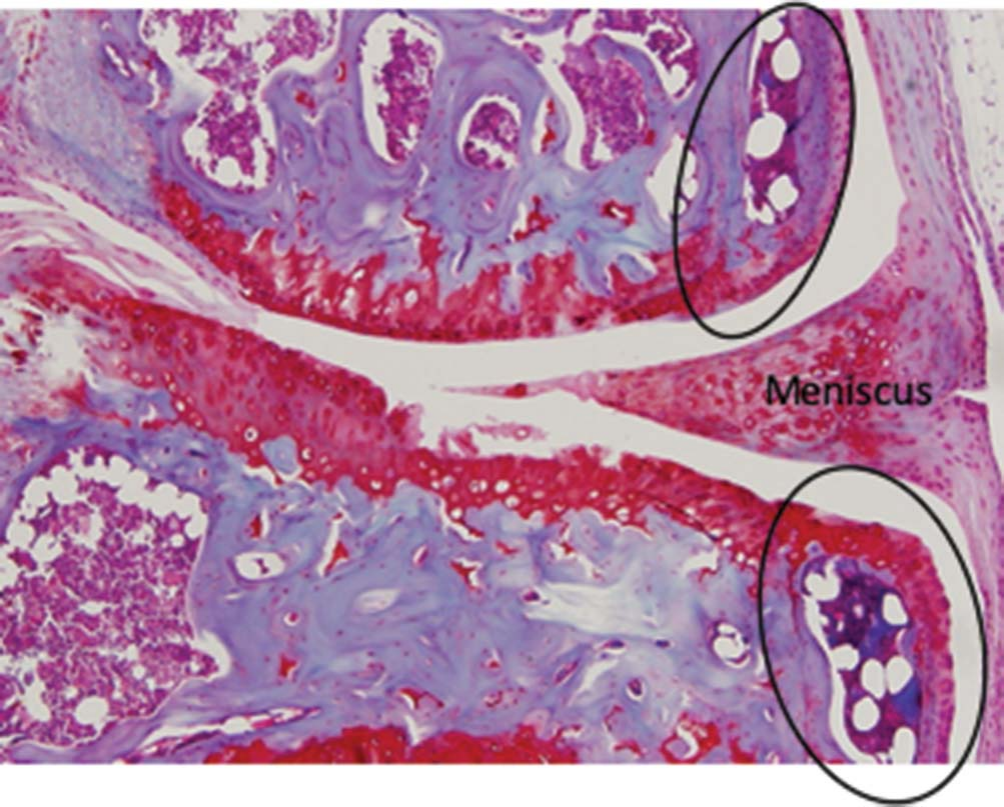
Murine osteophyte 8 weeks after DMM showing medial compartment of the knee joint in coronal view. Note clear demarcation between old lateral boarder of epiphysis and new bone (osteophytes circled). Osteophytes have cartilaginous caps that seem to “extend” the articulating surface of the joint. Moderate cartilage damage is seen in the midportion of the weight-bearing region of the joint particularly on the tibial surface (×40). DMM, destabilisation of the medial meniscus.

The bone is a very mechanoresponsive tissue and multiple changes occur during OA. New bone formation (osteophytosis) forms initially as a chondrophyte, and occurs at various sites in the joint, probably as a mechanoadaptive response.^32,94^ Osteophytes, often having a cartilaginous cap, change the overall shape of the articulating surface and have been shown to change in a consistent manner over time in age-related and posttraumatic human OA.^9^ In the mouse after joint destabilisation, the osteophyte has the appearance of extending the articulating surface (Fig. 2). Osteophytes in certain anatomical sites may be painful, for instance in the spine where they can restrict the exit of the nerve root.^68^ Whether they are painful without inducing nerve stretch or compression is less clear despite the fact that they are highly innervated (unlike the articular cartilage). Osteophytosis forms one component of the K&L score. In a carefully controlled study in which only patients with unilateral OA were examined so that the noninvolved joint could act as an intrapatient control, painful episodes did correlate with K&L score, and this was shown to be upheld when just joint space narrowing was considered but was lost when the bone changes were examined on their own.^74^ Three extrapolations can be drawn from this study: first, X-ray bone changes (osteophytosis, subchondral bone sclerosis, and cysts) are not principal drivers of OA pain; second, person-specific factors, such as central sensitisation, contribute greatly to the patient pain experience; and third, joint space narrowing (cartilage loss) does show an association with OA pain once these patient factors are controlled for.

Sclerosis of the subchondral bone plate occurs immediately below the articular cartilage especially on the damaged or most loaded side of the joint and this is also where bone marrow (BM) oedema develops. Bone marrow oedema is not visible on the plain X-ray but is visualised by MRI. Similar to results in the synovium, BM oedema shows weak but significant correlation with pain severity^20^ and there is also some evidence that prospective change in BM oedema correlates with a change in pain^30,84^ and the development of bone cysts.^13^

A few molecular studies have tried to examine these pathological processes and link them to pain. Kuttapitiya et al.^52^ performed an analysis in which they analysed regions of BM oedema (by MRI), taken from individuals undergoing joint replacement for severe symptomatic OA, and compared these with normal subchondral bone controls. A microarray analysis identified a number of molecules and pathways that were regulated specifically in the oedematous regions. By pathway analysis, these included molecules involved in neurogenesis, angiogenesis, and connective tissue remodelling. No evidence of *NGF* was found, but one of the most strongly regulated molecules was stathmin 2, known to be involved in regulating responsiveness to NGF.^42^

In mice, osteophytes form extremely rapidly (within 2 weeks) upon joint destabilisation and continue to grow slowly over time.^18^ The formation of the osteophyte does not seem to correlate with the onset of spontaneous pain-like behaviour (Fig. 1A). In one of our studies, we examined the differential expression of 67 molecules known to regulate pain. These included inflammatory cytokines, chemokines, neurotrophins, and their receptors. We microdissected the tissues from the mouse knee joint at the start of late OA pain behaviour and compared the expression levels with sham-operated controls.^26^ Only 8 of the 67 molecules were regulated 8 weeks after partial meniscectomy compared with sham. When considering the whole joint RNA, these included *Ngf*, as we had seen previously after DMM, bradykinin receptors (*Bdkr*) B1 and B2, tachykinin (*Tac1*), tachykinin receptor (*Tacr1*), *Tnf*, *Trpv4*, and *Vegf* (the latter 3 being <1.5-fold). When microdissected tissues were analysed separately, *NGF, Bdkrb1, Tac1,* and *Tacr1* were regulated in the articular cartilage. *Bdkrb2* was regulated in the epiphysis (containing subchondral bone and osteophyte combined). Due to technical challenges, it was not possible at this time to exclude regulation of molecules in the synovium.^26^ We validated the results by repeating the study at the time of pain-like behaviour development in a different surgical OA model, DMM. The same molecules were regulated in the joint at the time of pain with some minor differences. Importantly, in both studies, *Ngf* was exclusively upregulated in the articular cartilage.^26^

## 8. The role of cartilage in osteoarthritis pain

Of all the tissues considered by rheumatologists, connective tissue experts, and pain biologists over the years, the articular cartilage has been at the bottom of the list as a candidate tissue for driving pain because it is aneural and insensate. Our results in the mouse were provocative and unexpected but seemed to suggest that damaged articular cartilage was capable of making pain-inducing molecules. This result is corroborated by the demonstration that *NGF* represents of 1 of 7 chondrocyte subsets recently identified by single-cell RNA sequencing of human OA articular cartilage^40^ and was identified in early microarrays studies of severely damaged OA cartilage.^87^ Simple mechanical injury of cartilage that activates inflammatory signalling within the chondrocyte by a process we have termed “mechanoflammation”^97^ also strongly induced *Ngf* (300-fold within 8 hours of injury).^26^ Injury was also able to upregulate *Tac1, Bdkr1,* and *Bdkr2*. Presented in a graphical abstract, we proposed a model by which cartilage damage, particularly towards the basal layer, causes upregulation and release of Ngf and other pain-inducing molecules, to sensitise local pain fibres and induce new sprouting and neoinnervation of the tissue.^98^ This theory fits with the observed neoinnervation of the osteochondral junction documented by Walsh in late human OA, which relates to pain severity,^5,101^ and supported by recent studies in murine OA in which new nerves develop in the subchondral bone late in disease, localised to the region below the most damaged cartilage.^79^ Moreover, in a recent article by Zhu et al., pain driven by new nerve growth within the subchondral bone is also supported. Here, the authors demonstrate that axonal elongation is dependent on netrin-1, secreted by osteoclasts, which facilitates the passage of the nerve as it extends through the bone. Deletion of netrin-1 in osteoclasts or early treatment with a bisphosphonate was able to prevent pain-like behaviour in OA mice.^113^ It is tempting to speculate that the osteoclast-dependent process is primarily concerned with the process of neoinnervation of the tissue but not beyond this period. Once the new nerve is established, osteoclast inhibition is less likely to modulate pain and this might explain why bisphosphonate trials in human OA seem to have at best a modest effect on symptoms and no effect on structural outcomes.^95,106^ NGF is possibly the directional cue for these new nerves, as has been demonstrated in tumours,^41^ and once formed it is also acts to sensitise them, thus explaining rapid clinical success in anti-NGF trials. New nerves require nutrients from new blood vessels and this would fit with the strong angiogenic signature found in BM lesions by microarray.^52^

## 9. Summary—the origin of peripheral osteoarthritis pain—a new model emerging

**Figure 3.**
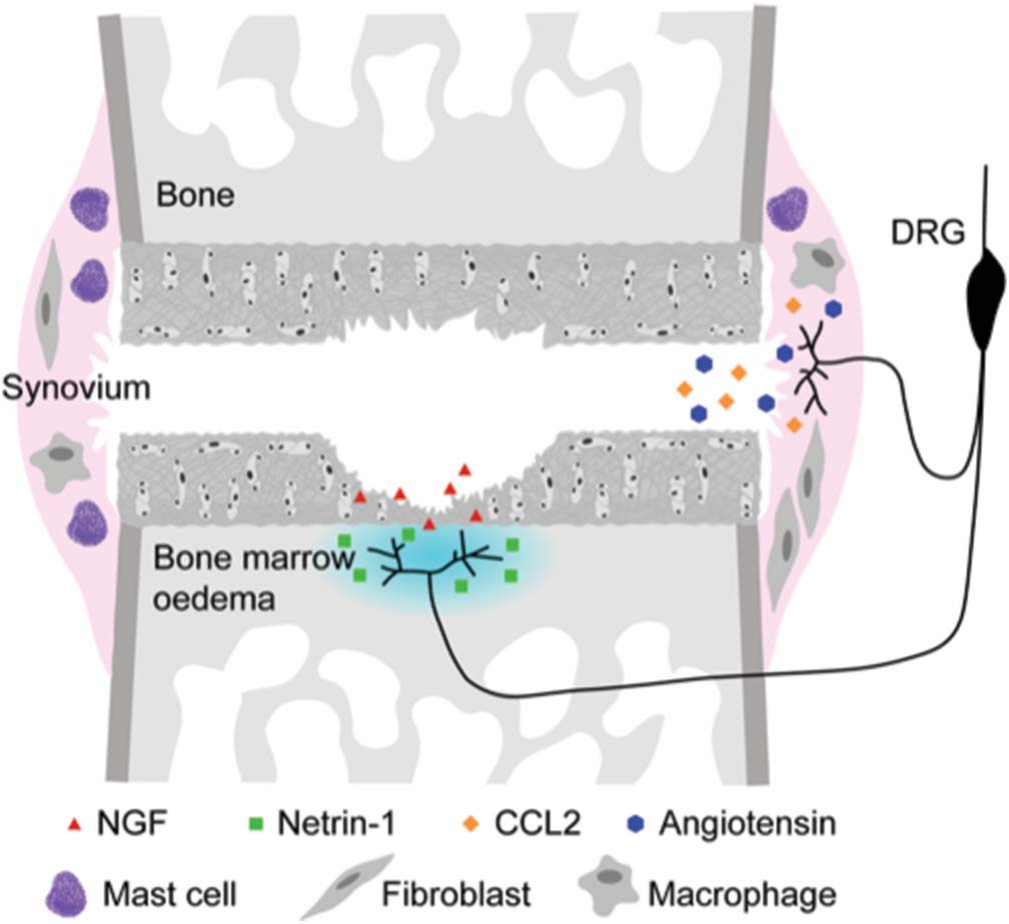
Proposed model for the molecular pathogenesis of pain in OA. Neoinnervation of the osteochondral junction is driven by NGF release from damaged cartilage and facilitated by osteoclast mediator netrin-1 within the oedematous bone marrow. Further sensitization within the joint is facilitated by CCL2, and likely other inflammatory mediators, eg, those released from mast cells. Angiotensinogen, processed by angiotensin-converting enzyme (ACE) to angiotensin (AT) II in the synovium, sensitises neurons through ATII type 2 receptor (AT2).

It has been fashionable in recent years to describe OA as a whole joint disease—this is hard to refute if the protagonists mean that most of the tissues in the joint are changed in disease. It has perhaps been less clear whether symptomatic disease, specifically pain considered here, is also driven by multiple tissue processes occurring in the joint at the same or different times and contributing to the pain experience. Nor has it been clear whether different joint pathologies could give rise to distinct pain phenotypes. The emerging literature would tend to suggest this, although there remain significant gaps in our knowledge. To date, the strongest evidence seems to lie within the subchondral bone where new blood vessels provide nutrient supply to axonal growth and neoinnervation of the osteochondral junction, likely driven by NGF released from basal articular chondrocytes (Fig. 3). Blockade of angiogenesis or axonal extension suppresses pain in experimental models consistent with this proposed process.^19,113^ The role of the synovium in driving pain is less clear. Despite strong correlative evidence from epidemiological studies and the experience in other synovitic diseases where targeting inflammation is efficacious, the synovium does not seem to be the principal pain driver in OA. The synovium is making some inflammatory molecules such as the chemokine CCL2, which leads to inflammatory response in the dorsal root ganglia of OA mice.^70^ Whether the synovitic component somehow primes and thereby augments the strong NGF-driven process, or whether it acts independently is as yet unknown. Either way, measurements of such molecules could help to phenotype patients and provide a rationale for stratification in the management of OA pain.

## Conflict of interest statement

In the past 3 years, T.V. has been on Advisory Boards for UCB, GSK, and Mundipharma. She directs the STEpUP OA Consortium, which receives financial project support (no personal gain) from Galapagos, Fidia, and Samumed. She has no other conflicts of interest to declare.
